# Non-Myopic Foveomacular Retinoschisis: Stellate Non-Hereditary Idiopathic Foveomacular Retinoschisis (SNIFR) and Central Anomalous Retinoschisis with Mid-Peripheral Traction (CARPET)

**DOI:** 10.3390/diagnostics16091285

**Published:** 2026-04-24

**Authors:** José Mª Ruiz-Moreno, Margarita Zamorano, Mariluz Puertas, Jorge Ruiz-Medrano

**Affiliations:** 1 Department of Ophthalmology, Puerta de Hierro-Majadahonda University Hospital, 28222 Madrid, Spain; margazamo@gmail.com (M.Z.); jorge.ruizmedrano@gmail.com (J.R.-M.); 2 Ocular Microsurgery Institute (IMO), 28035 Madrid, Spain; 3 Department of Ophthalmology, Castilla-La Mancha University, 02008 Albacete, Spain; 4 Medical School, Universidad Autónoma de Madrid, 28049 Madrid, Spain

**Keywords:** stellate non-hereditary idiopathic foveomacular retinoschisis, SNIFR, central anomalous retinoschisis with mid-peripheral traction, CARPET, vitreoretinal traction

## Abstract

**Background and Clinical Significance:** To describe two cases within the spectrum of non-myopic foveomacular retinoschisis, including stellate non-hereditary idiopathic foveomacular retinoschisis (SNIFR) and central anomalous retinoschisis with mid-peripheral traction (CARPET), and to highlight the role of multimodal imaging in identifying vitreoretinal traction in their pathogenesis and management. **Case Presentation:** *First Case Report*: A 57-year-old man presenting with bilateral visual decline. Multimodal imaging, including spectral-domain and en face optical coherence tomography (OCT), demonstrated characteristic features of SNIFR, with schisis at the Henle fibre layer and outer plexiform layer and persistent posterior hyaloid adhesion. Medical treatment was ineffective. Over two years, complete posterior vitreous detachment occurred, followed by spontaneous anatomical resolution of the schisis and full visual recovery. *Second Case Report*: A 63-year-old man with severe unilateral visual loss. Imaging revealed marked mid-peripheral vitreoretinal traction extending toward the posterior pole; associated with foveoschisis, central neurosensory detachment, and an outer lamellar macular hole, consistent with CARPET syndrome. The patient underwent pars plana vitrectomy with traction release. Postoperatively, complete anatomical resolution of both macular and peripheral schisis was achieved, with partial visual recovery. **Conclusions:** These cases support vitreoretinal traction as an important pathogenic mechanism in selected forms of non-myopic foveomacular retinoschisis. SNIFR may resolve spontaneously following posterior vitreous detachment, whereas CARPET represents a more severe tractional phenotype that may require surgical intervention. Careful multimodal imaging assessment of the vitreoretinal interface is essential for accurate diagnosis and management. These findings further characterise CARPET and expand the clinical spectrum of traction-related non-myopic foveomacular retinoschisis.

## 1. Introduction

Non-myopic foveoschisis comprises a rare and heterogeneous group of macular disorders characterized by splitting of the retinal layers at the fovea in the absence of high myopia or posterior staphyloma. Although foveoschisis is classically associated with myopic tractional maculopathy, increasing recognition of non-myopic forms has broadened the differential diagnosis of foveomacular retinoschisis maculopathy and highlighted the importance of multimodal imaging.

Stellate non-hereditary idiopathic foveomacular retinoschisis (SNIFR) was first described by Ober et al. in 2014 [1]. It is characterized by schisis at the level of the Henle fibre layer (HFL) and outer plexiform layer (OPL), producing a typical spoke–wheel pattern on correctly segmented en face OCT. SNIFR has traditionally been considered idiopathic, non-hereditary and relatively benign, often affecting middle-aged women with preserved visual acuity. However, subsequent reports have demonstrated a frequent association with peripheral retinoschisis and incomplete posterior vitreous detachment (PVD), suggesting that vitreoretinal traction may contribute to its pathogenesis.

In 2025, Feo et al. described a more severe phenotype of SNIFR termed Central Anomalous Retinoschisis with Mid-Peripheral Traction (CARPET) [2]. This entity is characterized by central neurosensory detachment (NSD), an outer lamellar macular hole (OLH), and marked mid-peripheral vitreoretinal traction.

To date, there is no clear consensus on the optimal management of SNIFR and CARPET syndrome. We report two cases within this spectrum that may help guide clinical decision-making: bilateral SNIFR with spontaneous resolution after complete posterior vitreous detachment, and CARPET requiring vitrectomy with peeling of mid-peripheral vitreoretinal traction. To our knowledge, this is the second published report of CARPET, and our findings provide additional clinical and imaging characterisation while highlighting practical implications for management of these conditions.

## 2. Case Report

### 2.1. Case 1

A 57-year-old man was referred to our clinic with a history of gradually progressive bilateral visual decline over several months. He described mild blurring of central vision in both eyes, without metamorphopsia, photopsia, or acute visual changes. His past ocular history was notable only for myopic LASIK (−4.00 dioptres) performed 20 years earlier, with stable refractive outcomes thereafter. He denied any history of systemic disease, inflammatory conditions, retinal dystrophies, ocular trauma, or exposure to medications associated with retinal toxicity. The patient’s refraction at presentation was +2.00/−0.25 at 180° in the right eye and +2.00/−0.75 at 40° in the left eye.

On presentation, best-corrected visual acuity (BCVA) was 20/32 in the right eye and 20/40 in the left eye. Intraocular pressure measured 15 mmHg bilaterally. Slit-lamp examination of the anterior segment was unremarkable, with clear lenses and no signs of inflammation. Dilated fundus examination revealed a bilateral stellate appearance at the macula, characterized by subtle radial striations and a slightly blunted foveal reflex. Optic nerve head appearance was normal, without glaucomatous excavation, making glaucoma-related schisis unlikely.

No optic disc pit, coloboma, epiretinal membrane, or signs of posterior staphyloma were identified. The absence of posterior staphyloma, together with the patient’s refraction and clinical history, makes a myopic schisis unlikely.

**Figure 1:**
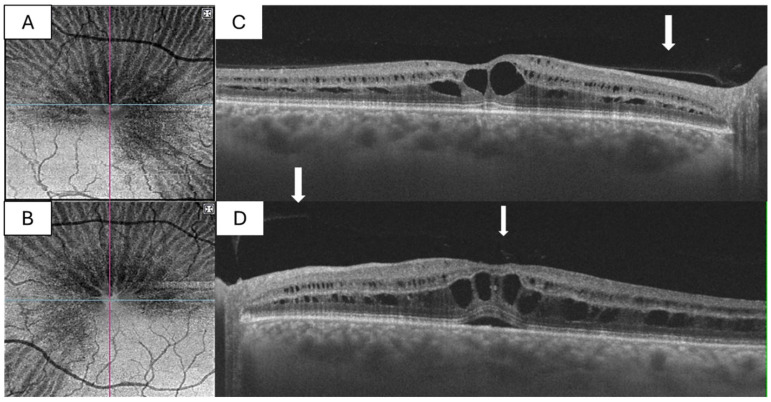
(**A**,**B**): “En face” OCT images (deep retinal) revealing the characteristic spoke–wheel configuration in right eye and left eye. (**C**,**D**): Cross-sectional OCT demonstrating intraretinal schisis localized to the Henle fiber layer, with persistent posterior hyaloid adhesion at the macula (arrow).

Spectral-domain OCT demonstrated bilateral foveomacular retinoschisis with splitting predominantly at the level of the Henle fibre layer (HFL) and outer plexiform layer (OPL). Hyporeflective schitic cavities were present centrally, with preservation of the outer retinal layers and ellipsoid zone. There was no evidence of full-thickness macular hole, neurosensory detachment, or frank vitreomacular traction. En face OCT with careful deep-layer segmentation at the HFL/OPL level revealed a characteristic spoke–wheel configuration centred on the fovea. OCT angiography demonstrated flow voids corresponding to the schitic spaces, without abnormal vascular proliferation or leakage (Figure 1). Full-field electroretinography was within normal limits, supporting exclusion of inherited retinal dystrophy.

Given the preserved visual acuity and absence of overt traction on cross-sectional imaging, conservative medical therapy was initially attempted. The patient received topical nepafenac for three months without anatomical or functional improvement. This was followed by dorzolamide 2% twice daily, again without measurable change in OCT appearance or visual acuity. A trial of topical corticosteroids and sub-Tenon triamcinolone injection was subsequently performed, with no significant response. In view of the lack of benefit and stable vision, treatment was discontinued and the patient was monitored with serial OCT examinations.

**Figure 2:**
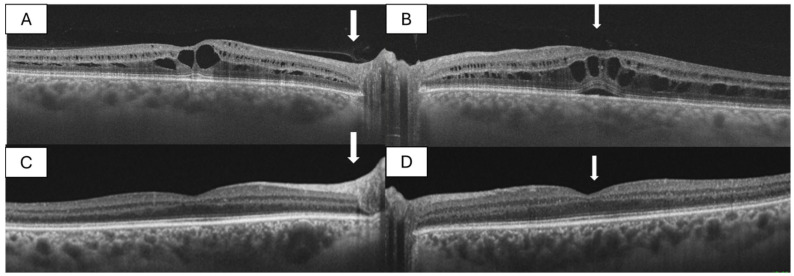
(**A**,**B**): Cross-sectional OCT showing intraretinal schisis localized to the Henle fiber layer and outer plexiform layer, persisting despite treatment with dorzolamide, topical corticosteroids, and sub-Tenon triamcinolone. (**C**,**D**): Cross-sectional OCT images showing complete resolution of intraretinal schisis and full posterior hyaloid detachment. The arrow indicates the posterior hyaloid, which is attached in the upper images and detached in the lower images.

Over a two-year follow-up period, gradual spontaneous improvement was observed. BCVA improved to 20/20 in both eyes. Follow-up OCT demonstrated complete resolution of the schitic cavities and restoration of normal foveal contour. Importantly, a complete posterior hyaloid detachment was documented in both eyes at the time of anatomical resolution (Figure 2), suggesting a temporal relationship between vitreous separation and recovery.

### 2.2. Case 2

A 63-year-old man presented with a two-week history of decreased central vision in the left eye. He reported blurred vision without pain, photopsia, or prior similar episodes. He had no known systemic disease and no previous ocular history. The right eye was asymptomatic.

At presentation, BCVA in the left eye was 20/400, while the right eye measured 20/20. Intraocular pressure was within normal limits in both eyes. Anterior segment examination was unremarkable. Fundus examination of the left eye showed subtle neurosensory macular detachment without haemorrhages, exudates, or optic disc anomalies. The peripheral retina demonstrated areas suggestive of vitreorretinal traction.

**Figure 3:**
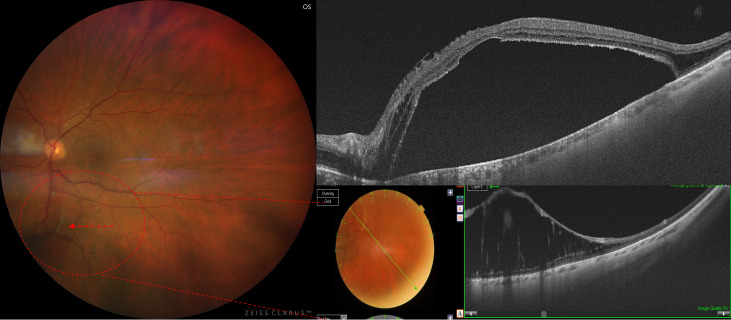
Widefield colour fundus photograph showing mid-peripheral traction in the inferior retina (dashed circle). The inferior scan highlights tractional elevation of the retina with schitic changes. Cross-sectional OCT images demonstrate marked mid-peripheral vitreoretinal traction extending towards the posterior pole, associated with central neurosensory detachment, outer retinal schisis and outer macular hole. These findings are consistent with Central Anomalous Retinoschisis with Mid-Peripheral Traction (CARPET). The red arrow indicates the mid-peripheral vitreoretinal traction extending towards the retina and posterior pole.

Multimodal imaging revealed marked mid-peripheral vitreoretinal traction arising from the inferior retina and extending towards the fovea. Widefield OCT confirmed peripheral retinoschisis contiguous with areas of vitreoretinal adhesion. Macular OCT demonstrated central neurosensory detachment, splitting of the outer retinal layers consistent with foveoschisis, and the presence of an outer lamellar macular hole. The ellipsoid zone appeared disrupted centrally. These findings were consistent with the recently described CARPET phenotype (Central Anomalous Retinoschisis with Mid-Peripheral Traction) (Figure 3).

Given the severity of anatomical disruption, the presence of neurosensory detachment and lamellar hole, and the marked visual impairment, surgical intervention was recommended. The patient underwent pars plana vitrectomy with induction of posterior hyaloid detachment and release of the mid-peripheral vitreoretinal traction. Internal limiting membrane peeling was not performed. A thorough central and peripheral vitrectomy was completed, followed by 20% SF6 gas tamponade. During induction of the posterior hyaloid detachment, an inferior juxtapapillary retinal tear occurred and was promptly treated with endolaser photocoagulation. No primary retinal breaks were identified intraoperatively.

**Figure 4:**
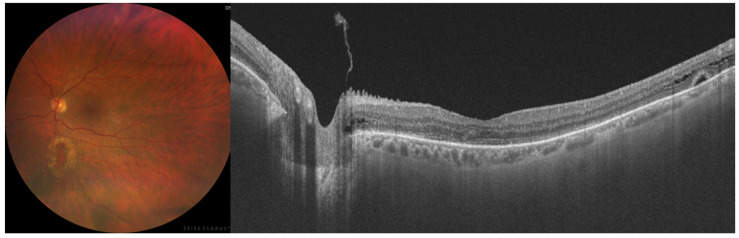
Widefield colour fundus photograph and macular OCT demonstrating complete resolution of the neurosensory retinal detachment and retinoschisis, with residual photoreceptor layer alterations and release of the inferior mid-peripheral traction.

At three months postoperatively, BCVA improved to 20/63. OCT confirmed complete resolution of the foveoschisis and neurosensory detachment, with restoration of the foveal contour. The associated peripheral retinoschisis had also resolved. Although outer retinal irregularity persisted, there was clear anatomical improvement corresponding with functional recovery (Figure 4). Further follow-up is planned, with the next examination scheduled at 3 months. Subsequent visits will be progressively extended to 6 and 12 months provided that visual acuity and macular OCT findings remain stable.

## 3. Discussion

Non-myopic foveomacular retinoschisis is a uncommon clinical finding with a broad differential diagnosis. Before considering SNIFR or related traction-mediated phenotypes, it is essential to exclude conditions capable of producing a macular star or schisis-like cavities through exudative, ischemic, or congenital mechanisms.

Exudative and inflammatory conditions must first be ruled out. Neuroretinitis remains one of the most important differential diagnoses, given its classic macular star configuration. However, unlike SNIFR, neuroretinitis is characterized by optic disc edema, true lipid exudation, and angiographic leakage [3,4]. The absence of disc swelling and the angiographically silent nature of schisis cavities in SNIFR are key distinguishing features. Furthermore, systemic infectious or inflammatory associations frequently accompany neuroretinitis but are absent in SNIFR and CARPET.

Malignant hypertension represents another critical masquerader. Bilateral macular star formation with cotton wool spots, retinal hemorrhages, and arteriolar narrowing should prompt urgent systemic evaluation [5]. Blood pressure measurement is advisable in any patient presenting with stellate maculopathy. The absence of vascular leakage and systemic hypertensive signs in our cases supports the diagnosis of non-exudative tractional schisis.

Hereditary retinoschisis, particularly congenital X-linked retinoschisis (CXLR), must also be excluded. CXLR typically presents in younger male patients with bilateral involvement and a characteristic electronegative ERG pattern [6]. In contrast, SNIFR usually affects older individuals, frequently women, and demonstrates normal ERG findings. Genetic testing is negative in SNIFR.

Structural causes such as myopic tractional maculopathy, optic disc pit maculopathy, and glaucoma-associated macular schisis should also be considered. By definition, non-myopic foveoschisis excludes high axial myopia and posterior staphyloma. Optic disc pit maculopathy can produce intraretinal schisis and subretinal fluid, but careful optic nerve head examination typically reveals the pit, and fluorescein angiography may show late leakage [7]. Glaucomatous macular schisis is associated with optic nerve cupping and visual field defects. None of these features were present in our cases.

Drug-induced cystoid macular edema, particularly related to niacin or taxane chemotherapy, may mimic angiographically silent cystic changes; however, these entities typically involve the inner nuclear layer and lack the characteristic spoke–wheel HFL splitting seen in SNIFR [8]. Detailed medication history is therefore indispensable.

SNIFR was first described by Ober et al. [1] in a series of 22 eyes from 17 patients with foveomacular retinoschisis in the absence of any identifiable hereditary or acquired cause, introducing the term stellate nonhereditary idiopathic foveomacular retinoschisis (SNIFR). The condition was predominantly observed in female patients (94%), frequently associated with mild to moderate myopia (72%) without a hereditary pattern and not associated with high or pathological myopia, was unilateral in the majority of cases (70%) and regarded as an idiopathic and largely non-progressive macular disorder.

SNIFR is diagnosed when a radial spoke-like pattern is seen around the fovea and OCT shows splitting at the level of the Henle fibre layer, after other causes of retinoschisis have been ruled out. The characteristic spoke–wheel pattern of SNIFR becomes evident only when the segmentation slab is positioned at the level of the Henle fibre layer and outer plexiform layer. Superficial segmentation or automated default settings may obscure this configuration, potentially leading to misinterpretation as nonspecific cystoid macular edema. Careful deep-layer segmentation should therefore be regarded as a diagnostic key feature.

Although historically SNIFR described as idiopathic and non-progressive, increasing evidence supports a traction-mediated mechanism. Multiple series have demonstrated a high prevalence of incomplete or anomalous posterior vitreous detachment (PVD) in affected eyes [9,10,11]. These findings suggest that chronic, low-grade vitreoretinal interface forces may induce splitting at structurally susceptible retinal layers.

Bloch et al. reported that up to 86% of eyes with SNIFR showed persistent hyaloid adhesion or incomplete PVD [12], suggesting that acquired biomechanical forces may contribute to foveomacular schisis. In their series, visual acuity was generally preserved and remained stable. Other reports have described anatomical improvement following spontaneous or surgically induced posterior hyaloid separation [13,14,15], further supporting the tractional hypothesis.

Last year Feo et al. described the long-term evolution of SNIFR associated with mid-peripheral retinoschisis (MPRS) and identified three patterns: centripetal progression towards the fovea, spontaneous regression, or stability [2]. Their longitudinal multimodal imaging analysis suggested that incomplete or anomalous PVD, particularly when associated with persistent mid-peripheral vitreoretinal adhesion, may represent a common pathogenic denominator linking MPRS and SNIFR. Importantly, They also introduced a new syndrome known as CARPET, characterized by central NSD, OLH, and marked mid-peripheral traction. This phenotype appeared to represent a traction-amplified form of SNIFR, in which progressive mid-peripheral forces extend centripetally, eventually compromising outer retinal integrity at the fovea. Notably, surgical release of mid-peripheral traction in one CARPET case resulted in complete anatomical resolution and marked visual improvement, whereas untreated cases demonstrated short-term anatomical and functional deterioration, presumably due to progressive tractional forces.

The Henle fiber layer likely represents a structural weak point within the foveal microarchitecture. Its oblique photoreceptor axonal orientation predisposes it to shearing stress when exposed to anteroposterior or tangential traction [16]. Importantly, these forces may be diffuse or peripherally anchored rather than focal, explaining why obvious vitreomacular traction is frequently may remain unrecognized on OCT without careful vitreomacular assessment. Widefield OCT and careful inspection of the vitreoretinal interface and mid-periphery are therefore essential. The frequent coexistence of peripheral retinoschisis further supports a shared biomechanical substrate. It is conceivable that mid-peripheral vitreoretinal adhesions act as anchoring points, transmitting centripetal forces toward the macula. In CARPET, these forces appear sufficiently intense to disrupt the outer retina, resulting in NSD and OLH. The presence of neurosensory detachment in this context implies a more profound mechanical disturbance, potentially compromising the outer blood–retinal barrier and leading to secondary photoreceptor dysfunction.

Furthermore, hyporeflective cystic spaces predominantly confined to the Henle fibre layer, as observed in our case, have been described more frequently in traction-related macular oedema, whereas exudative processes typically involve multiple retinal layers, including both the inner nuclear layer (INL) and the HFL–outer nuclear layer complex [17]. Although minimal cystic changes were also present within the INL in our patient, these were limited in extent and did not display the multilayered petaloid configuration characteristic of exudative macular oedema. Importantly, our case highlights that the presence of small inner nuclear layer cysts should not preclude the diagnosis of SNIFR when the overall imaging pattern, absence of angiographic leakage, and—most notably—the complete anatomical resolution following posterior vitreous detachment are strongly suggestive of a traction-mediated mechanism. In addition, dysfunction of Müller cell hydro-ionic regulation has been hypothesised as a contributing factor in the development of such cystic spaces within structurally vulnerable regions of the fovea [18].

To date, different therapeutic approaches have been described although no consensus exists regarding SNIFR optimal treatment. As vision is usually well preserved in SNIFR, invasive treatment to release vitreomacular adhesion is rarely needed. Pharmacologic intervention with carbonic anhydrase inhibitors has yielded variable outcomes. Although Ajlan et al. reported anatomical and functional resolution in one patient treated with topical dorzolamide [19], most published cases have not demonstrated significant benefits. The use of topical non-steroidal anti-inflammatory drugs (nepafenac) and corticosteroids has been reported to address a potential inflammatory component contributing to retinal fluid accumulation, while sub-Tenon triamcinolone has been employed to provide a stronger and more sustained anti-inflammatory effect [20]. However, reported outcomes have been inconsistent, with variable therapeutic response across published cases. Other therapeutic approaches, such as anti-VEGF injections have not demonstrated clear benefit in SNIFR and therefore are generally not recommended.

In the first case, bilateral SNIFR resolved spontaneously after complete PVD, with full anatomical and functional recovery. Importantly, this occurred despite the absence of response to medical therapy. This temporal relationship between PVD completion and anatomical resolution suggests a possible role of vitreoretinal adhesion in the persistence of schisis cavities rather than intrinsic retinal degeneration or idiopathic cause.

In contrast, the second case fulfilled the criteria for CARPET and showed marked focal mid-peripheral traction associated with NSD and an outer lamellar macular hole. Although vitrectomy led to complete anatomical resolution of both macular and peripheral schisis, visual recovery was only partial. The different functional outcomes between the two cases are likely related to differences in the severity and structural impact of traction rather than duration. In SNIFR, traction was probably milder and did not cause significant outer retinal disruption. In CARPET, the presence of NSD and OLH suggests greater mechanical stress on the photoreceptors.

These contrasting clinical courses suggest that SNIFR and CARPET have been proposed to represent different manifestations within a possible traction-mediated spectrum. At one end lies classic SNIFR, characterized by relatively mild interface forces and preserved outer retinal structure, often capable of spontaneous resolution following PVD completion. At the other end lies CARPET, in which amplified mid-peripheral traction generates sufficient stress to induce neurosensory detachment and lamellar macular hole formation, with greater risk of irreversible photoreceptor disruption.

To our knowledge, this is only the second published report of CARPET. The resolution of both central and peripheral schisis following surgical release of traction highlights a potential role of mid-peripheral vitreoretinal forces in this phenotype. While our case demonstrates that early surgical intervention may be considered in selected patients to prevent anatomical progression and possible irreversible outer retinal damage, these findings should be interpreted cautiously given the very limited evidence currently available.

Management should be guided by careful multimodal image assessment of the vitreoretinal interface. In typical SNIFR with stable vision, observation with serial OCT may be appropriate, particularly when vitreous separation is evolving. If, however, visual acuity declines and traction persists, vitrectomy may be considered. In contrast, CARPET features such as central NSD, OLH, marked traction and visual decline may justify earlier surgical intervention to reduce the risk of permanent outer retinal damage.

The concept of a SNIFR/CARPET disease spectrum has been previously proposed in the literature, with CARPET suggested as a more severe manifestation within the SNIFR spectrum. As the definition of CARPET is still evolving, these observations should be considered provisional. However, given the limited number of reported cases, further studies including larger case series are required to better define the potential relation between these entities.

## 4. Conclusions

In conclusion, our cases support vitreoretinal traction as an important mechanism in selected forms of non-myopic foveomacular retinoschisis, particularly SNIFR and CARPET. Accurate diagnosis requires careful exclusion of exudative, hereditary, and structural conditions, as well as proper deep-layer en face OCT segmentation to reveal the characteristic spoke–wheel pattern. Recognizing SNIFR and CARPET is important for prognosis and management, especially to differentiate stable cases that can be observed from traction-dominant forms that may require surgical treatment.

## Data Availability

The original contributions presented in this study are included in the article. Further inquiries can be directed to the corresponding author.

## Abbreviations

The following abbreviations are used in this manuscript:

SNIFR	Stellate non-hereditary foveomacular retinoschisis
CARPET	central anomalous retinoschisis with mid-peripheral traction
PVD	Posterior vitreous detachment
NSD	Neurosensory detachment
BCVA	Best-corrected visual acuity
HFL	Henle fibre layer
OPL	outer plexiform layer
OCT	optical coherence tomography
